# The impact of family and peer relationships on developmental trajectories of depressive and anxiety symptoms among young people: a person-oriented approach

**DOI:** 10.1186/s13034-026-01033-4

**Published:** 2026-02-11

**Authors:** Benti G. Buli, Peter Larm, Kent W. Nilsson, Cecilia Åslund, Fabrizia Giannotta

**Affiliations:** 1 Division of Public Health, School of Health, Care and Social Welfare, Malardalen University, Box 883, Västerås, Sweden; 2 Department of Public Health, Stockholm University, SE-106 91, Stockholm, Sweden; 3 Centre for Clinical Research, Uppsala University, Västmanland County Hospital Västerås, S-72189 Västerås, Sweden; 4 Department of Medical Sciences, Child and Adolescent Psychiatry, Uppsala University, Uppsala, Sweden; 5 Division of Public Health, School of Health, Care and Social Welfare, Malardalen University, Box 883, Västerås, Sweden; 6 Department of Medical Sciences Child Health and Parenting (CHAP), Department of Public Health and Caring Sciences, Uppsala University, Uppsala, Sweden; 7 Department of Psychology, University of Turin, Via Verdi 10, 10124 Turin, Italy

**Keywords:** Depressive symptoms, Anxiety, Trajectory, Social relationships, Adolescents, Group-Based Trajectory Modeling

## Abstract

Adolescent mental health is a growing global concern, with depressive and anxiety symptoms on the rise over recent decades. The significance of supportive social relationships, particularly within family and peer groups, is well-established in research. However, limited evidence exists on the impact of social relationships in predicting the developmental trajectories of mental health problems over time. This study aims to fill this gap by identifying groups with distinct trajectories of depressive and anxiety symptoms among adolescents and exploring the impact of various factors, including family relationships, peer relationships, sex, and country of origin, on predicting individuals' likelihood of belonging to specific trajectories. Data come from a community sample of Swedish young people born in 1997 and 1999 (N = 1834, 50% female) and followed over 12 years (four waves conducted at three-year intervals). The Group-Based Trajectory Modeling (GBTM) was used to identify profiles of young people longitudinally. Moreover, multinomial logistic regression models were conducted to examine the predictive effects of social relationships, sex and country of origin on depressive and anxiety symptom trajectories. Four groups were identified for both depression (Stable-Low, Declining, Rising-D, Persistent High) and anxiety (Low and Declining, Stable-Moderate, Rising-A, High). Positive social relationships with family and peers emerged as robust predictors across depressive and anxiety symptom trajectories. Female participants consistently exhibited higher mean scores of depressive and anxiety symptoms than males, while participants originating from countries outside the Nordic region were at higher risk of belonging to risky depressive symptom trajectory groups. These findings highlight the critical importance of strengthening supportive family and peer environments for preventive strategies and targeted interventions in adolescent mental health.

**Supplementary Information:**

The online version contains supplementary material available at 10.1186/s13034-026-01033-4.

## Introduction

Adolescent mental health is a pressing global concern [1, 2], with its prevalence rising steadily over recent decades [3–9]. Amidst this concerning trend, the significance of supportive social relationships, particularly within family and peer groups [10, 11], cannot be overstated. Research indicates that positive family relationships with children and adolescents, characterized by positive parenting styles including warmth [12, 13], structure [14–17], and autonomy support [18, 19], contribute significantly to adolescents' mental well-being. This aligns with the buffering hypothesis where social support can help protect individuals from the negative impacts of stressful life events [10, 11]. Conversely, negative familial interactions, such as rejection and coercion, may exacerbate mental health problems [20, 21].

Moreover, peer relationships play a pivotal role in adolescent development. Supportive peer relationships serve as protective factors against mental health problems [22], while negative interactions, like peer victimization, can have detrimental effects [23]. Positive peer relations, especially in the context of school environments, as crucial social and academic arenas [24], are associated with reduced mental health risks [25]. In a similar vein, a study revealed that family and peer supportive relationships, taken together, are among the most important protective factors for adolescent mental health, outweighing other influences such as the school environment or individual traits [26]. This is explained by Cohen and Wills [11] social buffering hypothesis which suggests social support moderates the impact of stress on mental health. When adolescents encounter stressors, strong family and peer relationships reduce the likelihood of developing depression or anxiety.

Although associations between social relationships and mental health outcomes are well-established [22, 23, 26, 27], significant questions remain about the predictive role of these relationships over time. Few studies, such as those by Chen and Harris [26] and Finan et al. [28], have examined whether social relationships in adolescence can predict changes in mental health problems from adolescence into adulthood. However, these studies largely relied on methodologies that model variability around a single population mean trajectory. Such mean-based approaches, while informative, have been criticized for oversimplifying developmental patterns by masking individual differences and failing to capture the complexity of heterogeneous developmental pathways [29, 30].

In contrast, person-centered methods—such as group-based trajectory modeling—are built on the assumption that populations consist of distinct subgroups, each following a unique trajectory. These methods have been shown to yield more accurate and meaningful insights into developmental processes [31, 32], making them especially suited for exploring the interplay between social relationships and mental health outcomes.

Furthermore, individual-level factors well known to influence mental health, such as sex differences [33, 34] and country of origin [35], deserve further investigation in this context. Understanding how these characteristics interact with social relationships to predict membership in distinct trajectory groups can provide critical knowledge for designing targeted interventions and advancing preventive mental health strategies.

To address these gaps, this study followed a community sample of adolescents into adulthood, using four waves of data in order to identify groups with distinct trajectories of depressive and anxiety symptoms from adolescence to early adulthood. Moreover, it explores the impact of protective social factors, such as positive family and peer relationships in adolescence, alongside individual factors, including sex and country of origin, as well as cohort, on predicting the likelihood of individuals belonging to specific trajectories. In other words, the study hypothesizes that supportive social relationships and individual characteristics jointly influence the likelihood of belonging to specific mental health trajectories. We selected sex, country of origin, and cohort as predictors because these demographic variables are consistently identified as key stratifiers of adolescent mental health. Sex differences are well-documented, with girls showing higher prevalence and distinct developmental patterns of depression and anxiety [26]. Country of origin similarly shapes exposure to stressors such as discrimination and cultural adaptation, which influence mental health trajectories [27]. Cohort membership was included to account for contextual differences across time [28]. These variables therefore provide a robust basis for examining heterogeneity in trajectories while allowing us to isolate the impact of supportive social relationships.

## Methods

### Participants and data collection

In this study, participants were young individuals born in 1997 and 1999, all residing in the Västmanland region of Sweden. The region’s population is considered fairly representative of Sweden, reflecting national distributions of education, employment, ethnic background, and income levels, as well as a mix of urban and rural settings [29]. Data collection was conducted in four rounds. Initial enrollment occurred in 2012 (T1) when participants were 15 and 13 years old, respectively, as part of the 'Survey of Adolescent Life in Västmanland Cohort' (SALVe cohort), where data were collected from 1834 individuals. Subsequent surveys occurred at three-year intervals, with the fourth wave occurring in October 2021. During these subsequent surveys, data were collected from 1643 individuals in 2015 (T2), 1212 in 2018 (T3), and 1067 individuals in 2021 (T4). 50% (N = 534) of the final sample were females. For more comprehensive details, additional information can be obtained from Vadlin, Åslund [30] and Mohamed, Rukh [31].

### Measurements

#### Depressive symptoms

The Depression Self-Rating Scale—A criterion (DSRS-A) [32] was used to measure depressive symptoms among the participants. The scale was constructed from 15-item questions presented to each participant, asking if they had experienced any depressive feelings during the 2 weeks prior to the survey date. The 15 items were transformed into nine clinical symptom groups using the DSRS-A, as outlined in the Fourth Edition of the Diagnostic and Statistical Manual of Mental Disorders (DSM-IV), with each symptom group coded as either 0 if no symptoms or 1 if they had at least one symptom. The mapping of the 15-item questionnaire into a 9-item scale is presented in Supp. Table S1. The scale created from the summation of the nine clinical symptoms resulted in scores ranging from 0 to 9, with higher scores indicating a greater severity of symptoms. Cronbach’s alpha was 0.79 at T1, 0.82 at T2, 0.80 at T3, and 0.85 at T4. Because Cronbach’s alpha yields estimate comparable to other reliability tests, such as Kuder–Richardson Formula 20 (KR-20), which are recommended for categorical items [33], no additional test was conducted. We have also conducted configural and metric measurement invariance tests to assess whether loadings were comparable across groups and sex [34]. The results presented in Supp. Table S2 indicate these assumptions were supported.

#### Anxiety symptoms

Two versions of scales were used to measure anxiety symptoms. At T1 and T2, when the mean ages of the participants were 14.4 and 17.4, respectively, the generic Spence Children’s Anxiety Scale (SCAS) [35] was used. At T3 and T4, when the mean ages of the participants were 19.9 and 22.9 years, respectively, the shorter version Adult Anxiety Scale (AAS-15) [36] was used. The SCAS comprised 44 questions, six of which were filler questions and 38 used to construct the scale, with response categories ranging from 0 to 3. The AAS, however, had 15 questions, but with the same range of responses as SCAS. The total indices of the scales therefore ranged from 0 to 114 (38 x (0 to 38) × 3) for SCAS and 0–45 (15 x (0 to 15) × 3) for AAS. The scales showed strong internal consistency with McDonald’s Omega [37]of 0.97 at T1, 0.94 at T2, 0.93 at T3, and 0.93 at T4.

#### Positive family relationship

Positive parent child relationship was measured at T1 using three subscales of the adolescent version of the Parents as Social Context Questionnaire (PASCQ) developed by Skinner, Johnson [38], and validated using the same dataset this study utilizes [39]. These subscales assess three positive dimensions of parenting, namely Warmth (e.g., "My parents are always glad to see me"), Structure (e.g., "When I want to do something, my parents show me how"), and Autonomy Support (e.g., "My parents trust me"). We then constructed a composite parent’s supportive relationship variable by summing the scores of these three styles. The resulting scale has scores ranging from 0 to 36, with a higher score indicating a more positive parenting style.

#### Positive peer relationship

The relationship an adolescent may have with peers or age-mates in their surroundings was measured at T1 based on three questions: (1) Do you find it easy or difficult to make new friends? (2) Do you have any close friends in your school class? (3) Do you have any close friends in your free time/in your residential area? For the first question, alternative responses were: very difficult (0), difficult (1), easy (2), and very easy (3). For the second and third questions, the alternative responses include: no (0), yes, one (1), yes, 2 (2), and yes, 3 or more (3). A composite variable was created by summing these scores, resulting in a scale with scores ranging from 0 to 16, with a higher score indicating a stronger peer relationship.

#### Country of birth

Information on country of birth was obtained at T1 from the national population registry. Country of birth was coded as 1 if the adolescent and at least one parent were born in Sweden or another Nordic country, and 0 otherwise.

#### Sex

Sex was coded as boys = 0, girls = 1. Sex was not self-report but was based on the personal identification number retrieved at T1. In Sweden, sex is assigned at birth and recorded in the national population registry. Each individual is assigned a personal identification number, in which certain digits correspond to the registered sex (female or male).

#### Cohort

As described above, this study utilized data collected from two cohorts of individuals born in 1997 and 1999 in Västmanland. The 1997 birth cohort was coded as 1, while the 1999 birth cohort was coded as 0.

### Statistical analysis

Longitudinal data provide an empirical foundation for analyzing developmental trajectories across time [40]. Traditional statistical approaches often emphasize individual variability around the mean, which may obscure meaningful heterogeneity in patterns of change [41]. In reality, distinct subgroups within a population frequently follow different developmental trajectories, reflecting diverse pathways of change [42]. Group-Based Trajectory Modeling (GBTM) offers a robust framework to identify and capture these subgroup trajectories, enabling researchers to examine changes over time in a more nuanced and person-centered manner. A drawback of GBTM is the occurrence of within-group zero variance, which can be addressed by minimizing the number of trajectory classes specified in the model [43].

GBTM in STATA software was employed to identify the developmental trajectory groups of depressive and anxiety symptoms among young individuals in the sample obtained from the SALVe cohort. We estimated two separate models, one for depression and one for anxiety, and included covariates individually in each model to assess their unique effects.

We adopted a data-driven approach in our developmental trajectory analysis to empirically identify heterogeneity in mental health trajectories and allow the data to reveal distinct developmental patterns [44, 45]. To reduce the risk of overfitting and capitalizing on chance, we applied established model selection criteria, including the Bayesian Information Criterion (BIC), entropy, and substantive interpretability, and examined trajectory stability. Accordingly, finite mixture models [45] were employed to determine the optimal number of distinct trajectory groups for individuals in relation to their experiences of depressive and anxiety symptoms. We used the Zero-Inflated Poisson (ZIP) model, incorporating combinations of linear, quadratic, and cubic polynomials, to estimate the depressive trajectories, given that the responses were inflated with zero values. For anxiety trajectories, we used the Censored Normal (cnorm) model, chosen due to the shape of the distribution [45], with combinations of linear, quadratic, cubic, and quartic polynomials. The Bayesian Information Criterion (BIC), with a value closest to zero, and the absence of a group with fewer than 5% of the total sample members, were utilized to select the best model with the most suitable number of groups (refer to Supp. Table S3). Subsequent to fitting the group-based models, goodness-of-fit tests were conducted using the average posterior probability (APP) with a cutoff point of > 70%, Odds of Correct Classification (OCC) with a cutoff point of < 5.0, and observed probability (≥ 5% of total probability) to assess the likelihood that an individual with a specific profile belongs to a particular trajectory group [45] (refer to Supp. Table S4). The role of factors including sex, age, cohort, country of birth, and relationship with family and friends in predicting group membership was examined using multinomial logistic regression analysis. Relative risk (RR) was used to measure the effect of each independent variable on the probability of being in any of the trajectory groups at significance level less than 0.05.

#### Missing data handling

Missing data on the longitudinal outcome variables were handled using the maximum likelihood estimation procedure implemented in group-based trajectory modeling, which allows inclusion of all available data under the Missing at Random (MAR) assumption. To assess the plausibility of the MAR assumption and evaluate potential attrition effects, we examined whether missingness across the four waves was associated with baseline covariates or with observed values of the outcome variables. We additionally compared the mean levels of the key outcomes, depressive symptoms and anxiety symptoms, between participants assessed only at T1 (T1-only) and those who continued participation in subsequent waves (T1 & T2, T1 & T3, and T1 & T4). Group mean differences were tested using z-tests with a significance level of 0.05. No significant associations or group differences were found, suggesting that attrition was not systematically related to the main study variables. Therefore, the trajectory models were estimated using all available outcome data without imputation. We did not correct for multiple testing in our attrition-bias analyses because doing so could mask imbalances that are small but meaningful [46].

### Ethical considerations

Standard ethical procedures, by Swedish Law, Ethical Review Act 2003: 460, have been diligently adhered to, and approval was secured by the Ethical Review Board in Uppsala (Dnr. 2012/187). Consent has been acquired from all participants and, where applicable, from their parents in the case of underage individuals. Records of those who did not grant consent for the use of their data have been systematically excluded from the analysis. For instance, one record at wave 1 and seven records at wave 4 were omitted from the analysis due to lack of consent.

## Results

The study results revealed a moderate level of dropout across the waves of data collection. Among the 1834 participants assessed at T1, 10% (N = 191) dropped out by T2, 34% (N = 622) by T3, and 42% (N = 767) by T4. Further analysis of the attrition rate, as presented in Table 1, indicated no significant difference in mean scores of both depressive and anxiety symptoms between T1-only participants and those who participated in T1 and subsequent waves.

**Table 1:** Comparison of mean scores of depressive and anxiety symptoms between participants in wave 1 (T1) only and those continuing assessment across subsequent waves (T2, T3, or T4)

	T1 & subsequent waves		Participated in T1 only		Mean difference	95% CI of mean difference	
	Mean	SD	Mean	SD		Lower	Upper
Depressive Symptoms							
T1 versus T2	1.85	2.18	2.10	2.51	0.25	− 0.13	0.63
T1 versus T3	1.83	2.13	2.10	2.51	0.27	− 0.11	0.65
T1 versus T4	1.80	2.14	2.10	2.51	0.30	− 0.08	0.71
Anxiety symptoms							
T1 versus T2	0.709	0.28	0.72	0.29	0.009	− 0.04	0.06
T1 versus T3	0.710	0.28	0.72	0.28	0.008	− 0.04	0.06
T1 versus T4	0.713	0.28	0.72	0.28	0.005	− 0.04	0.05

*CI* Confidence Interval, *SD* Standard deviation, *T1* Time 1 (baseline), *T2* Time 2, *T3* Time 3, and *T4* Time 4

### Descriptive analysis of depressive and anxiety symptoms across key variables

Table 2 presents the distribution of mean scores for depressive symptoms and anxiety symptoms across different independent variables. Female participants consistently exhibited significantly higher mean scores for both depressive and anxiety symptoms across all waves of the study. The 1997 cohort displayed significantly higher mean scores for depressive symptoms compared to the 1999 cohort only at T1 and T2, while no significant difference was observed in the mean scores of anxiety symptoms between the cohorts.

**Table 2:** Mean scores and standard errors of a) depressive symptoms and b) anxiety symptoms at T1, T2, T3, and T4 by main independent variables

a) Depressive symptoms								
	T1		T2		T3		T4	
	Mean (SE)	Z (p)	Mean (SE)	Z (p)	Mean (SE)	Z (p)	Mean (SE)	Z (p)
Female	2.23 (0.03)	16.94 (0.00)	3.55 (0.03)	31.95 (0.00)	3.64 (0.04)	24.19 (0.00)	3.80 (0.04)	19.02 (0.00)
Male	1.44 (0.04)		1.92 (0.04)		2.20 (0.05)		2.58 (0.03)	
Non-Nordic	2.09 (0.05)	4.59 (0.00)	3.25(0.05)	7.38 (0.00)	3.15 (0.06)	0.98(0.33)	3.59 (0.07)	3.65 (0.00)
Nordic origin	1.82 (0.03)		2.80 (0.03)		3.08 (0.03)		3.31 (0.03)	
1997—cohort	2.34 (0.33)	15.64 (0.00)	3.11 (0.03)	8.99 (0.00)	3.14 (0.04)	1.60 (0.11)	3.35(0.06)	0.51 (0.61)
1999—cohort	1.51 (0.33)		2.67 (0.04)		3.05 (0.04)		3.38 (0.04)	
b) Anxiety symptoms								
Female	0.79 (0.03)	3.75 (0.00)	0.91 (0.03)	5.59 (0.00)	1.00 (0.04)	6.33 (0.00)	1.07 (0.04)	5.41 (0.00)
Male	0.61 (0.04)		0.63 (0.04)		0.62 (0.05)		0.72 (0.05)	
Non-nordic	0.74 (0.05)	0.70 (0.49)	0.82 (0.05)	0.66 (0.51)	0.80 (0.07)	− 1.00 (0.32)	0.88 (0.07)	− 0.92 (0.36)
Nordic	0.70 (0.03)		0.79 (0.03)		0.87 (0.03)		0.96 (0.04)	
1997—cohort	0.73 (0.03)	0.86 (0.39)	0.79 (0.04)	− 0.04 (0.97)	0.87 (0.04)	0.58 (0.56)	0.91 (0.04)	− 1.23 (0.22)
1999—cohort	0.69 (0.03)		0.79 (0.04)		0.84 (0.04)		0.98 (0.04)	

*SE* Standard error, *z* z-statistic, *p* p-value, *T1* Time 1 (baseline), *T2* Time 2, *T3* Time 3, and *T4* Time 4

Participants originating from outside the Nordic region had significantly higher mean scores for depressive symptoms at T1, T2, and T4, whereas no significant difference was observed in anxiety symptom mean scores across the countries of origin (refer to Table 2 for detailed data).

### Depressive symptoms trajectories

**Fig. 1:**
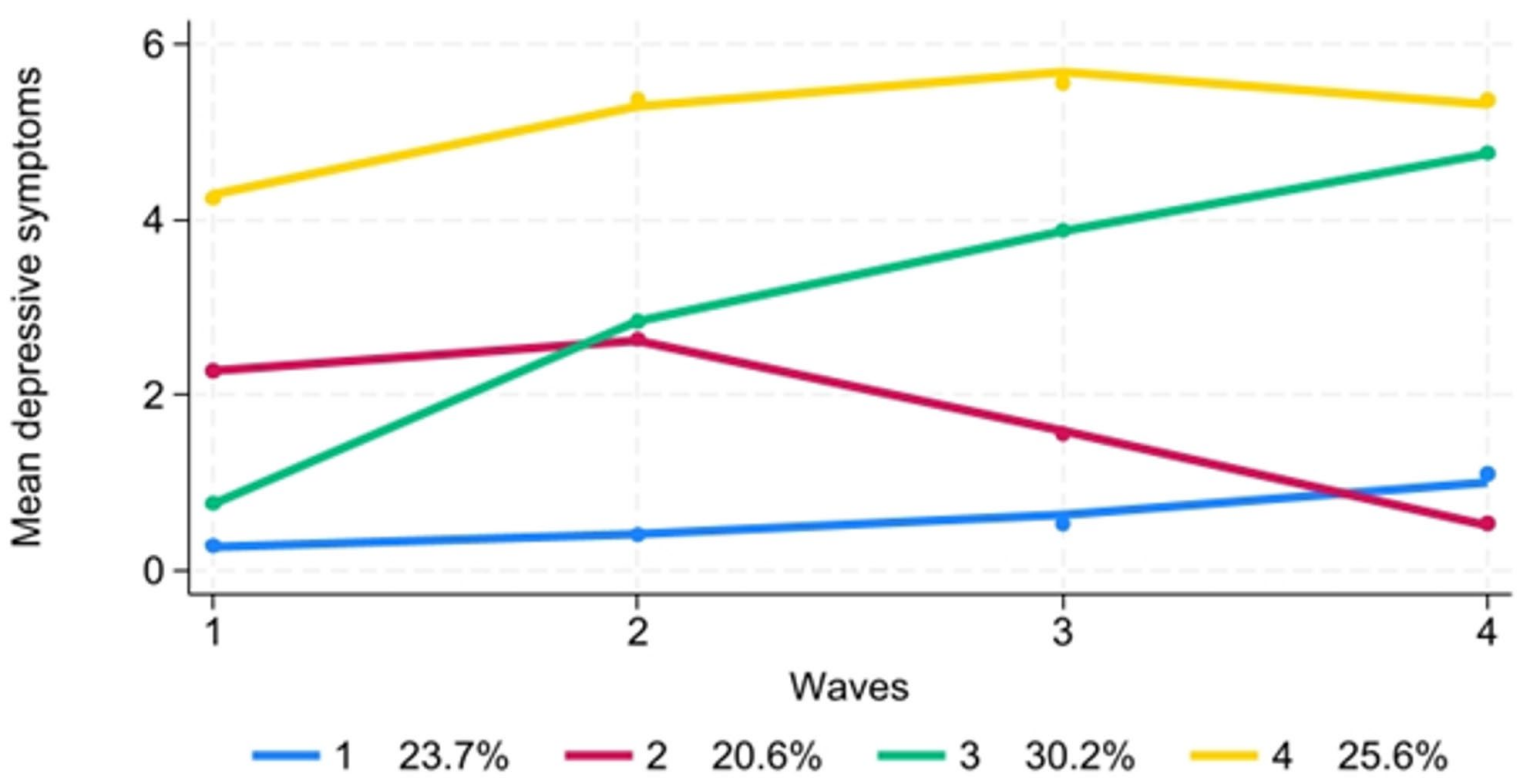
Trajectories of depressive symptoms among young people who were born in 1997 and 1999 in Vastmanland Region of Sweden. (Note: The number of observations for each trajectory group is reported in Supplementary Table S3. Group names: 1 = Stable-Low, 2 = Declining, 3 = Rising-D, and 4 = Persistent High)

The iterative process of identifying an appropriate number of trajectory groups for individuals yielded a four-group model based on the Bayesian Information Criterion (BIC) and the additional criterion of ensuring that no group comprised less than 5% of the total sample (see Fig. 1). The four trajectory groups are outlined as follows: (1) Stable-Low (23.7%): This group exhibits relatively low levels of depressive symptoms across the waves, with a slight and steady increase from T2 onwards. (2) Declining (20.6%): In this group, there is a relatively stable pattern of moderate depressive symptoms across waves, with a slight increase observed between T1 and T2, followed by a decline afterward. (3) Rising-D (30.2%): This group demonstrates a steady increase in depressive symptoms from T1 to T2, followed by continued increase afterward but at a slower rate. (4) Persistent High (25.6%): This group displays a sharp increase in depressive symptoms from T1 to T2, followed by a high and stable pattern, then a gradual decrease afterward.

### Anxiety symptoms trajectories

**Fig. 2:**
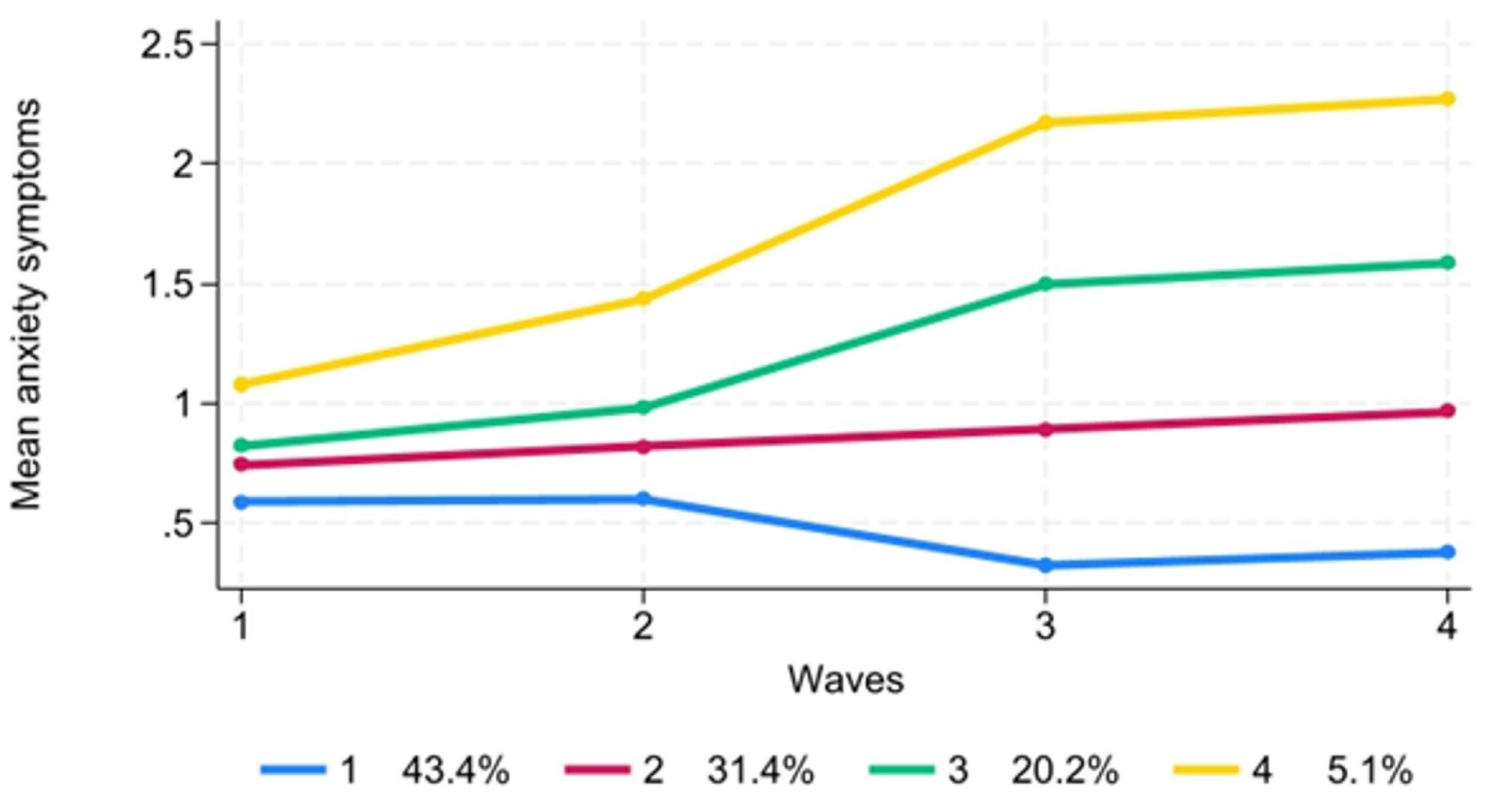
Trajectories of anxiety symptoms among young people who were born in 1997 and 1999 in Vastmanland Region of Sweden. (Note: The number of observations for each trajectory group is reported in Supplementary Table S3. Group names: 1 = Low and declining, 2 = Stable-moderate, 3 = Rising-A, and 4 = High)

Figure 2 presents the four trajectory groups of anxiety symptoms established based on the criteria discussed above. These groups include: (1) Low and Declining (43.4%): This group shows a low and stable pattern between T1 and T2 and a gradual decrease in mean anxiety symptoms from T2 to T3, followed by a slight rise afterward. (2) Stable-Moderate (31.4%): This group exhibits a relatively stable pattern of mean anxiety symptoms across waves, with a slight increase from T2 onwards. (3) Rising-A (20.2%): This group displays a steady increase in mean anxiety symptoms from T1 to T3, followed by a slower rate of increase afterward. (4) High (5.1%): This group demonstrates a sharp increase in mean anxiety symptoms from T1 to T3, followed by stable and high scores afterward.

###  Predictors of membership in trajectory groups

Table 3 presents predictors of membership in depressive and anxiety symptom trajectory groups. Strong family and peer relationships were consistently protective, reducing the likelihood of belonging to higher-risk trajectories for both depression and anxiety. Female adolescents had markedly higher odds of being in rising or high symptom trajectories compared to males, with the most pronounced effect observed for high anxiety symptoms. Nordic origin was associated with a lower likelihood of membership in depressive symptom trajectories, while no significant association was found for anxiety trajectories. Finally, adolescents born in 1997 were more likely than those born in 1999 to belong to rising or persistent high depressive symptom trajectories, whereas year of birth showed no clear association with anxiety trajectories.

**Table 3:** Multinomial logistic regression results of the association between trajectory groups of depressive symptoms (a) and anxiety symptoms (b) and predictors of memberships into specific trajectory groups

Depressive Symptoms (a)^†^	Declining	Rising-D	Persistent high
	RR (SE)	RR (SE)	RR (SE)
Family Relationship	0.89 (0.02) ***	0.78 (0.02) ***	0.86 (0.02) ***
Positive peer relationship	0.97 (0.04)	0.77 (0.04) ***	0.88 (0.04) **
Sex (Female versus male)	4.65 (0.72) ***	18.30 (3.56) ***	3.66 (0.63) ***
Country of origin (Nordic)	0.53 (0.11) **	0.42 (0.09) ***	0.36 (0.08) ***
Cohort of 1997 versus 1999	0.91 (0.14)	2.47 (0.42) ***	2.66 (0.45) ***

^†^*Reference group: Stable-Low*			
Anxiety symptoms (b)^††^	Stable-Moderate	Rising-A	High
	RR (SE)	RR (SE)	RR (SE)
Family Relationship	0.98 (0.01)	0.92 (0.01) ***	0.85 (0.02) ***
Positive peer relationship	0.82 (0.03) ***	0.75 (0.03) ***	0.64 (0.04) ***
Sex (Female vs. male)	8.12 (1.14) ***	12.66 (2.29) ***	88.58 (54.13) ***
Country of origin (Nordic)	0.87 (0.14)	1.19 (0.23)	1.19 (0.41)
Cohort of 1997 versus 1999	1.21 (0.16)	0.83 (0.13)	0.70 (0.19)

^††^Reference group: Low and declining

***P < 0.001, **P < 0.01, *RR* Relative Risk, *SE* Standard Error

## Discussion

This study followed young people in a county of Sweden who were born in 1997 and 1999, starting from when they were 15 and 13 years old, up to the age of about 24 and 22 respectively, to assess group developmental trajectories of depressive and anxiety symptoms. Five trajectory groups each for depressive symptoms and anxiety symptoms were tested, and finally, models with four distinct groups each were identified [45].

The four-trajectory group solution of depressive symptoms is consistent with findings from previous studies [47–49], with a slight difference in the shapes of the trajectories. These four trajectory groups seem to follow two different patterns where at T2, when the participants were at about the age of 16 and 18, the Persistent High and Declining groups started to stabilize and decline, respectively, whereas the Stable-Low and Rising-D groups continued to increase. The first pattern is consistent with previous studies that reported an increase in depressive symptoms from childhood through adolescence before decreasing in adulthood [50, 51]. The other two groups that increased from T2 onwards are also consistent with a study that reported higher scores of depressive symptoms post-adolescence than during or pre-adolescence periods [52]. Our study, however, differs methodologically from those by Ge, Natsuaki [50] and Natsuaki, Biehl [51], which based their trajectory modeling on age as the unit of analysis. Instead, our approach is based on individuals' mental health profiles at baseline, grouping those with similar profiles together. In this sense, our study contributes to literature by supporting the notion that a large group may consist of distinct subgroups of people with similar profiles [45, 53]. Our model also somehow resembles the three-group model solution by Coryell, Mills [54], which reported two decreasing and another persistently high depressive symptom trajectory groups. The group with rapidly falling and the other with persistently high trajectories are consistent with our Declining and Persistent High groups, respectively, while the difference in the rest may be attributed to the fact that Coryell, Mills [54] used a smaller sample size than ours and the age range was only between 15 and 20 years. Beyond methodological differences, variations in trajectory patterns may also reflect developmental transitions during adolescence. Neurobiological changes, such as heightened stress reactivity and ongoing maturation of emotion regulation systems, can amplify vulnerability to depressive and anxiety symptoms during mid- to late adolescence [55, 56].

Regarding the trajectories of anxiety symptoms, the four-trajectory groups solution with Low and declining, Stable-moderate, Rising-A, and High was partly consistent with findings from Spence, Lawrence [57], which reported a three-group model with stable-low, low-increasing, and high-decreasing trajectories. Like the stable-low group in Spence, Lawrence [57], which comprised most of the participants (89.6% male and 78.2% female), the first two trajectory groups in our findings comprise majority of the whole sample, following either a steadily declining or slowly increasing pattern. In the other two groups, Rising-A and High, however, there was a significant increase with age, especially from T2 onwards when the participants were 18 in the 1997 cohort and 16 in the 1999 cohort. This is consistent with McLaughlin and King [58], who found a decreasing trajectory of anxiety symptoms during adolescence, with exceptions for those with high levels of depressive symptoms. Therefore, in our case too, although the analysis of comorbidity is out of the scope of this study, the rise during late adolescence or early adulthood may be explained by the Rising-D and Persistent High depressive symptom trajectories in this study. Another study by Spielberg, Schwarz [59] explains the increase in anxiety symptoms from adolescence to early adulthood in three main ways. First, subthreshold anxiety during adolescence may progress to a diagnostic level in early adulthood, e.g. Zhong, Niu [60]. Second, pubertal testosterone may induce lasting changes in brain organization, resulting in weaker emotion regulation that may persist in adulthood, for example, Tyborowska, Volman [61]. Third, there may be distinct causal pathways for anxiety in adulthood, with anxiety in adolescence serving as a risk factor [62]. Thus, the increasing trajectory of anxiety symptoms observed in this study could be explained by any of these scenarios, in addition to possible explanation by co-morbidity with depressive symptoms in adolescence. In addition to biological factors, increasing academic demands, identity formation, and social role transitions during late adolescence may contribute to rising anxiety trajectories. These stressors interact with individual coping resources, potentially explaining why some adolescents experience escalating symptoms despite normative declines [63, 64].

One of the key findings of this study is the significant role of positive social relationships between adolescents and their families and peers in predicting group membership across various trajectories of depressive and anxiety symptoms. Increased positive relationships with family and peers was associated with a decreased risk of belonging to any trajectory group exhibiting higher levels of depressive or anxiety symptoms than the reference groups. These findings are consistent with existing evidence highlighting the beneficial impact of positive family relationships [65, 66] and positive peer relationships [22, 66] on mental health outcomes. Positive social relationships protect against worsening symptoms by providing crucial resources, as described in the social support or “buffer” hypothesis, which encompasses four distinct domains [10, 11, 22]. Emotionally, these relationships boost self-esteem and belonging, countering isolation and worthlessness that worsen anxiety and depression [67]. Informationally, they offer advice and guidance, enabling effective coping and reframing negative thoughts [11]. Tangible support addresses practical needs, reducing external pressures and freeing up mental resources [10, 67]. Finally, companionship provides shared activities and affection, combating loneliness and promoting well-being [11, 68] thereby reducing the likelihood of belonging to high-symptom trajectory groups. These findings underscore the critical role of supportive social environments, provided by both family and peers, in preventing the development of depressive or anxiety symptoms that adolescents often face, attributed to the enormous changes they undergo during adolescence [64]. Unique to this study is the revelation that family relationships serve as a robust predictor for individual membership within distinct groups exhibiting similar progressions of depressive and anxiety symptoms over time [45]. This uniqueness poses challenges in directly comparing our results with prior research; therefore, the findings need to be interpreted within this context. These findings can also be interpreted through attachment theory, which posits that secure relationships provide a foundation for emotional regulation and resilience [69, 70]. Similarly, Bronfenbrenner’s ecological systems theory emphasizes the interplay between family, peer, and broader social contexts in shaping mental health trajectories [71, 72].

Female participants consistently had higher mean scores of depressive and anxiety symptoms than their male counterparts throughout the waves. As a result, they were more likely than males to be placed in the trajectory groups other than the reference groups, that had lower mean scores of the symptoms. This is consistent with previous studies that reported higher depressive symptom trajectories with steeper increases among females than males [49, 73]. With regards to anxiety symptom trajectories, our findings were consistent with McLaughlin and King [58] only in that females had consistently higher mean scores of anxiety symptoms than males did, and hence the likelihood of being in the trajectory groups with higher levels of anxiety symptoms. In this study, sex was determined based on registry data, specifically the personal identification number, which reflects the sex assigned at birth. While this approach ensures objective and standardized classification, it does not account for gender identity, which may differ from assigned sex. Self-reported data, on the other hand, allow individuals to express their gender identity but may be influenced by social desirability bias or variability in how questions are interpreted. Future studies could benefit from integrating both registry-based and self-reported measures to better capture the complexity of sex and gender in relation to mental health outcomes. Beyond biological sex, gender socialization processes may partly explain these differences. Females often face greater interpersonal stress and societal expectations related to appearance and relationships, which can heighten vulnerability to internalizing symptoms during adolescence [74].

Finally, consistent with a previous study elsewhere that reported more depressive symptoms among ethnic minorities with persistent disparity over time [75], this study found that participants originating from countries outside the Nordic region were at higher risk of belonging to depressive symptom trajectory groups other than the reference groups. In Sweden, although existing studies show higher burdens of and increasing trends in depressive symptoms among adolescents with immigrant backgrounds [76], this study is the first, to our best knowledge, to investigate the impact of migration background on the developmental trajectory of this problem. These findings underscore the need for targeted mental health interventions that consider the unique vulnerabilities of adolescents with immigrant backgrounds. Understanding how migration influences the developmental course of depressive symptoms is crucial for designing culturally sensitive prevention and intervention strategies. Future research should explore the underlying mechanisms driving these disparities, including social determinants, acculturation stress, and access to mental health resources, to inform more effective policies aimed at promoting mental well-being among diverse adolescent populations. These disparities may be understood through acculturation stress theory, which suggests that navigating cultural adaptation, discrimination, and identity conflicts can increase psychological distress among immigrant adolescents, thereby influencing their symptom trajectories [77, 78].

This study, however, has some limitations. While in range with most community studies, this study faced a moderate attrition rate, potentially impacting the distribution of mean depressive and anxiety symptom scores between participants assessed at the initial time point (T1) and those who dropped out afterward. Noteworthy, we did not find relationships in the missing patterns in the outcome variables during subsequent measures. Furthermore, the missing pattern was unrelated to any of the main independent variables, such as family relationships, peer relationships, country of origin, and age. Although Group-Based Trajectory Modeling (GBTM) is a robust method for analyzing longitudinal data, it also has inherent limitations and assumptions. Variability in trajectory shapes and sizes, as well as potential model misspecification, could influence the accuracy and interpretation of the results [45]. Another limitation of this study is that we did not formally test longitudinal or group-based measurement invariance [79] for the latent construct of anxiety, due also to the fact that different scales were used at different time point. This decision was based on the notion that different forms of anxiety may manifest at different ages (for example, separation anxiety becomes less relevant in adulthood). Nevertheless, we used two versions of the measure that were validated with Swedish adolescents and adults [35, 36], which supports the reliability of the assessments. Nevertheless, caution is warranted when interpreting differences across time and subgroups. Furthermore, incorporating SES in future analysis would allow for a more detailed examination of its role in predicting both trajectory membership and the shape of developmental pathways. It is also imperative that future studies investigate interactions among predictors to clarify how their combined influence shapes anxiety and depression trajectories. Such analyses could reveal synergistic or buffering effects that are not apparent when factors are examined independently.

Nevertheless, the strengths of this study apparently outweigh the limitations. The GBTM allows for the examination of trajectory patterns of mean scores of depressive and anxiety symptoms among distinct groups within the sample. This methodological approach offers a nuanced understanding of how symptoms evolve over time and how different subgroups may experience varying trajectories [45]. By assessing the predictive effects of family and peer relationships on depressive and anxiety symptom trajectories, the study addresses a significant gap in literature. This comprehensive approach provides valuable insights into the role of social relationships in shaping mental health outcomes over time. To the best of our knowledge, this study is the first to investigate the predictive effects of family and peer relationships on depressive and anxiety symptom trajectories using the GBTM approach. While prior research has extensively documented the association between adolescent social support and later mental health outcomes, including depressive symptoms in adulthood [80, 81], most of these studies have relied on population-level averages. In contrast, GBTM enables a more nuanced understanding by identifying distinct symptom trajectories and examining how social relationships influence risk within specific subgroups [45]. This approach moves beyond headline findings to reveal differential patterns of vulnerability and protection, offering insights that can inform targeted interventions for adolescents at greatest risk.

### Conclusions

This study tracked adolescents in a Swedish county born in 1997 and 1999 from ages 15 and 13 to approximately 24 and 22, respectively, examining developmental trajectories of depressive and anxiety symptoms. The study identified four distinct trajectory groups for both depressive and anxiety symptoms, with patterns aligning partly with previous research but revealing unique nuances in trajectory shapes and progression. Especially, positive social relationships with family and peers emerged as significant predictors across these trajectories, consistent with existing evidence on their positive impact on mental health outcomes. These findings highlight the crucial role of social relationships in shaping the mental health trajectories of adolescents into early adulthood. From an intervention perspective, programs aimed at strengthening family bonds and fostering supportive peer networks could be particularly effective in mitigating depressive and anxiety symptoms over time, especially among early at-risk adolescents.

## Supplementary Information

Below is the link to the electronic supplementary material.

Supplementary Material 1.

## Data Availability

The datasets generated and/or analyzed during the current study are not publicly available due to Swedish privacy regulations but are available from the corresponding author on reasonable request.

## Declarations

### Generative AI and AI-assisted technologies in the writing process

During the preparation of this work, the first author used ChatGPT to check the language. After using this tool/service, the first author reviewed and edited the content as needed and takes full responsibility for the content of the published article.
