# Supplementary material for: The impact of family and peer relationships on developmental trajectories of depressive and anxiety symptoms among young people: a person-oriented approach
Source: Child Adolesc Psychiatry Ment Health. 2026 Feb 11;20:37. doi: 10.1186/s13034-026-01033-4 (PMC12997961; doi:10.1186/s13034-026-01033-4)
Supplement: Supplementary file 1 — Supplementary Material 1. [file 13034_2026_1033_MOESM1_ESM.docx]

# Supplementary materials

Supp. Table 1. Mapping a 15-item Depression Self Rating Scale for Adolescents (DSRS A) to a 9-item DSM IV Criterion based on Sund et al. (2001), Kroenke et al. (2001), and Sonnby et al. (2022)

| **DSM‑IV Criterion** | **A 15‑item Questionnaire** |
| --- | --- |
| Feeling down, depressed, or hopelessness | 1.Feeling depressed most of the day, nearly every day |
|  | 2. Feeling irritable most of the day, nearly every day |
| Little interest or pleasure in doing things | 3. Feeling much less interested than usual in most activities |
| Poor appetite or overeating | 4. Decreased appetite or unintentional weight loss of 2 kg or more |
|  | 5. Increased appetite nearly every day or weight gain of 2 kg or more |
| Sleep disturbance | 6. Poor nighttime sleep, difficulty falling asleep, frequent awakenings, or early morning awakening |
|  | 7. Increased need for sleep, sleeping longer in the mornings each day |
| Psychomotor changes | 8. Restlessness or difficulty sitting still |
|  | 9. Moving or speaking more slowly than usual |
| Fatigue | 10. Feeling tired or lacking energy most of the time, nearly every day |
| Guilt/worthlessness | 11.Feelings of worthlessness much of the time |
|  | 12. Recurring feelings of guilt about things done or not done |
| Concentration difficulties | 13. Had difficulty concentrating on tasks |
| Suicidal ideation | 14. Thoughts of being better off dead |
|  | 15. Thoughts of hurting or killing oneself |
| Kroenke, K., Spitzer, R. L., & Williams, J. B. (2001). The PHQ-9: validity of a brief depression severity measure. *J Gen Intern Med*, *16*(9), 606-613. <https://doi.org/10.1046/j.1525-1497.2001.016009606.x>  Sonnby, K., Skordas, K., Vadlin, S., Olofsdotter, S., Nilsson, K. W., & Ramklint, M. (2022). Psychometric validation of two versions of the adolescent Depression Self-Rating Scale (DSRS-A and DSRS-A Screener). *Nordic Journal of Psychiatry*, *76*(3), 233-242. <https://doi.org/10.1080/08039488.2021.1956583>  Sund, A. M., Larsson, B., & Wichstrom, L. (2001). Depressive symptoms among young Norwegian adolescents as measured by the Mood and Feelings Questionnaire (MFQ). *Eur Child Adolesc Psychiatry*, *10*(4), 222-229. <https://doi.org/10.1007/s007870170011> | |

Supp. Table 2. Longitudinal invariance of depression measure in all sample and across females and males

|  | **χ^2^** | **df** | **CFI** | **SRMR** | **RMSEA [90% CI]** | **Model comparisons** | | | |
| --- | --- | --- | --- | --- | --- | --- | --- | --- | --- |
|  |  |  |  |  |  | **Models** | **Δχ_SB_^2^** | **ΔCFI** | **ΔRMSEA** |
| **All Sample** | | | | | | | | | |
| Configural (M1) | 5971.173 | 66 | .999 | .015 | .011 [.000, .021] |  |  |  |  |
| Metric (M2) | 45.949 | 36 | .998 | .017 | .012 [.00, .021] | M2-M1 | 5925.224 (30)^***^ | -.001 | .006 |
| Scalar (M3) | 439.461 | 45 | .993 | .092 | .068 [.062, .064] | M3-M1 | 5531.712 (21)^***^ | -.006 | .057 |
| **Multigroup analysis for sex** | | | | | | | | | |
| Configural (M1) | 217.655 | 104 | .978 | .031 | .034 [.028, .041] |  |  |  |  |
| Metric (M2) | 242.720 | 112 | .975 | .034 | .036 [.029, .042] | M2-M1 | 25.05 (8)^***^ | -.003 | .002 |
| Scalar (M3) | 368.230 | 121 | .952 | .063 | .047 [.042, .053] | M3-M1 | 150.575 (17) ^***^ | -.026 | .032 |

Supp. Table 3. Bayesian Information Criterion (BIC), BIC factor and presence or absence of a group with <5% of total sample as criteria to select the best model.

| Number of trajectory groups | Depressive symptoms | | | Anxiety symptoms | | |
| --- | --- | --- | --- | --- | --- | --- |
|  | BIC* | BIC factor** | Group members <5% of total | BIC | BIC factor | Group members <5% of total |
| 1 | -12323.84 |  | No | -4053.12 |  | No |
| 2 | -11839.05 | 2.99 | No | -3145.87 | 3.26 | No |
| 3 | -11724.13 | 2.36 | No | -2948.39 | 2.60 | No |
| **4** | **-11692.63** | **1.80** | **No** | **-2867.18** | **2.21** | **No** |
| 5 | -11684.99 | 1.18 | No | -2845.08 | 1.65 | Yes |
| *BIC = Bayesian Information Classification; BIC factor = Log at base of 10 of the difference between BIC of group “n” and that of group “n-1” multiplied by 2 (BIC factor = Log_(B10)_(ΔBIC), where ΔBIC = BIC_n_ – BIC_n-1_). The models with best solution are presented in bold font. | | | | | | |

Supp. Table 4. Indicators of goodness of fit for trajectory models of depressive and anxiety symptoms.

| Trajectory Group | Number of members | Group APP | OCC | Occ PP | Observed Probability | Total Probability |
| --- | --- | --- | --- | --- | --- | --- |
| **Developmental trajectories of depressive symptoms** | | | | | | |
| Stable-Low | 447 | 0.847 | 17.974 | 19.161 | 0.235 | 0.224 |
| Declining | 347 | 0.766 | 14.706 | 13.892 | 0.182 | 0.191 |
| Rising-D | 597 | 0.795 | 8.481 | 8.802 | 0.314 | 0.306 |
| Persistent high | 408 | 0.874 | 25.306 | 23.787 | 0.215 | 0.225 |
| **Developmental trajectories of anxiety symptoms** | | | | | | |
| Low & declining | 707 | 0.809 | 7.154 | 7.807 | 0.372 | 0.352 |
| Satble-moderate | 411 | 0.806 | 15.104 | 14.603 | 0.216 | 0.222 |
| Rising-A | 589 | 0.737 | 6.236 | 5.995 | 0.310 | 0.318 |
| High | 92 | 0.863 | 124.275 | 110.080 | 0.051 | 0.054 |
| APP= Average posterior probability (>70%), OCC =odds of correct classification (based on maximum posterior probability of group membership (>5.0), OCC PP = odds of correct classification based on the weighted posterior probability; no group showed observed probability <5% of the total. | | | | | | |
